# Suppression of Retinal Neovascularization by Inhibition of Galectin-1 in a Murine Model of Oxygen-Induced Retinopathy

**DOI:** 10.1155/2017/5053035

**Published:** 2017-03-24

**Authors:** Ning Yang, Wenxi Zhang, Tao He, Yiqiao Xing

**Affiliations:** Eye Center, Renmin Hospital of Wuhan University, 238 Jiefang Road, Wuhan, Hubei Province 430060, China

## Abstract

Galectin-1 (Gal-1) has been proved to be an important factor in the process of tumor angiogenesis recently. As a small molecule, OTX008 serves as a selective inhibitor of Gal-1. In this study, the role of Gal-1 and the antiangiogenic effect of OTX008 on retinal neovascularization (RNV) were investigated using a mouse model of oxygen-induced retinopathy. The outcome indicated that Gal-1 was overexpressed and closely related to retinal neovessels in OIR. After intravitreal injection of OTX008 at P12, the RNV was significantly reduced at P17, measuring by cross-sectional H&E staining and whole-mount fluorescence. Our results demonstrate the inhibitory function of OTX008 on RNV, which provides a promising strategy of treating retinal angiogenic diseases such as retinopathy of prematurity and proliferative diabetic retinopathy.

## 1. Introduction

Retinal neovascularization (RNV) originates from the existing vessels of the retina, generally extending through the internal limiting membrane (ILM) and growing into the vitreous cavity [1]. RNV is a common feature of ischemic retinopathies, including retinopathy of prematurity (ROP), central retinal vein occlusion (CRVO), and proliferative retinopathy (PDR) [2–4]. A mouse model of oxygen-induced retinopathy (OIR), which is consistent and reliable at quantifying RNV, has been used extensively [5, 6]. Intravitreal injection of antivascular endothelial growth factor (VEGF) therapies is efficient in treating RNV, due to the dominating role of VEGF in angiogenic signaling [7, 8]. However, these anti-VEGF agents still carry some restrictions, as well as a risk of complications [9]. Additionally, the mechanisms of pathological RNV are not completely understood yet.

Galectin-1 (Gal-1), with a single carbohydrate recognition domain (CRD), was recently identified as a mediator in the process of tumor angiogenesis [10–12]. Gal-1 is involved in several biological actions, such as apoptosis, immune adjustment, cell proliferation, and adhesion [11, 13]. It was reported that Gal-1 and VEGF were separately regulated [14]. However, the role of Gal-1 in ocular neovascularization has not been deeply investigated previously. Several approaches of inhibiting Gal-1 were currently under research, such as blockade of CRD using oligosaccharides and specific monoclonal antibodies. However, their poor selectivity confined their applications [15]. OTX008 was lately reported as a novel calixarene compound which is derived from anginex and bonded to Gal-1 on the side back face, far away from the *β*-galactoside binding site [16–18]. OTX008 could especially and selectively interact with Gal-1 and restrain its functions [18]. Previous study showed that OTX008 could decrease Gal-1 protein level in SQ20B cells in a time-dependent manner [18]. It has been revealed that OTX008 inhibits the proliferation and migration of endothelial cells in vitro and tumor-related angiogenesis in multiple models [18]. However, the function of Gal-1 in OIR and the inhibitory effect of OTX008 in RNV were not clear yet.

In this study, we found that Gal-1 was overexpressed in OIR model. Intravitreal injection of OTX008 was used to inhibit the role of Gal-1. The results showed that OTX008 significantly reduced RNV in the OIR mouse model.

## 2. Materials and Methods

### 2.1. Animals

C57BL/6J mice were obtained from the Laboratory Animal Center of Wuhan University. All animal-related experiments in this study were performed in accordance with the guidelines of the ARVO statement for the Use of Animals in Ophthalmic and Vision Research. The experimental protocols were approved by the Committee on the Ethics of Animal Experiments of Wuhan University. All efforts were made to minimize the suffering of mice.

### 2.2. Oxygen-Induced Retinopathy and Intravitreal Injection

Mice were divided into four groups. About 25 to 30 mouse pups were used per group, and both eyes were removed for experimental analysis. Mice in the room air (RA) group were raised in room air for 17 days before being killed without any intervention. OIR model was induced based on well-established protocols [5, 6]. Newborn pups at postnatal day 7 (P7) were placed to 75% oxygen circumstance for 5 days in series. At P12, the mice were returned to room air for another 5 days until P17. At P12, both eyes of the mouse in the OIR-OTX008 group received 1 *μ*l of OTX008 (Quality Control Chemicals Inc., Walnut, CA, USA; 0.25 *μ*g/*μ*l, diluted in sterile PBS) through intravitreal injection, while mice in the OIR-PBS group were administered the same volume (1 *μ*l) of sterile PBS as a vehicle control. Intravitreal injection was proceeded as previously reported through microinjection system [19]. In short, after anesthesia, the micropipette was penetrated through the sclera at the level of the pars plana and angled avoiding the injury to the lens and the vessels. There was no obvious infection, toxicity, or inflammation related to intravitreal injection and dose used.

### 2.3. Confocal Laser Scanning Microscopy

Cryosections of the eyeballs were processed as previously reported [20]. Eyecups were immediately fixed in 4% paraformaldehyde (PFA) for about 30 min. After graded dehydration in 10% and 20% sucrose for 1 h, respectively, and then in 30% sucrose overnight at 4°C, the eyecups were embedded into the optimum cutting temperature medium (Sakura Finetek, CA, USA). The cryosections were cut vertically (12 *μ*m) using Leika CM1950 crostat (Leika, Wetzlar, Germany). The sections were mounted on polylysine slides and immunostained with the Gal-1 antibody (5 *μ*g/ml, R&D System, MN, USA) overnight at 4°C. After rinsing with PBS-T buffer (0.1% Triton-X 100), cryosections were then incubated with FITC-AffiniPure Donkey Anti-Goat IgG (1 : 200, Jackson ImmunoResearch Laboratories, PA, USA) for 1 h at room temperature. The images were taken by laser scanning microscopy (FV1200; Olympus, Tokyo, Japan).

### 2.4. Whole-Mount Fluorescent Staining

At P17, mice received intraperitoneal (IP) injections with 60 mg/kg pimonidazole HCL (HP, Hypoxyprobe Inc., Burlington, MA, USA), 90 min before being executed. HP was used as a novel biomarker to indicate the hypoxic condition of the retinas [21]. The retinal flat-mount staining and quantification analysis was conducted as previously described [6]. After disposal of the anterior segment and vitreous, enucleated eyes were fixed in 4% PFA for 1 h. Then, the retinas were microdissected and flattened with four radial incisions. After being rinsed and blocked, the retinas were incubated for 48 h at 4°C with Griffonia simplicifolia isolectin B4 (IB4) conjugated to Alexa Fluor 594 (1 : 200, Invitrogen/Thermo Fisher Scientific, MA, USA) and HP antibody (anti-pimonidazole rabbit antisera, 1 : 100, Hypoxyprobe Inc., Burlington, MA, USA); Gal-1 antibody (5 *μ*g/ml, R&D System, MN, USA) and neuropilin-1 (Nrp-1) antibody (10 *μ*g/ml, R&D System, MN, USA) were used to detect Gal-1's distribution and quantification of Gal-1 and Nrp-1. After rinsing with PBS-T 3 times, the retinas were then incubated with FITC-AffiniPure Goat Anti-Rabbit IgG (1 : 200, Jackson ImmunoResearch Laboratories, PA, USA) for 24 hours at 4°C; FITC-AffiniPure Donkey Anti-Goat IgG (1 : 200, Jackson ImmunoResearch Laboratories, PA, USA) was used for Gal-1 or Nrp-1. At last, the retinas were flat mounted in an antifade reagent on the slides. The images were captured by fluorescence microscope (BX63, Olympus, Tokyo, Japan). The quantification analysis of the retinal neovascularization (RNV), vaso-obliteration (VO), and hypoxic zones was performed as previous protocols [6, 22].

### 2.5. Hematoxylin and Eosin (H&E) Staining

Enucleated eyes were fixed in 4% PFA for 24 hours and embedded in paraffin. Serial sections (5 *μ*m) were sagittally cut parallel to the optic and then stained with H&E. Only sections through the optic nerve were chosen. Preretinal neovascular cell nuclei were quantified as previously reported [5, 19].

### 2.6. Western Blot Analysis

Western blot analysis was performed essentially according to previous protocols [19]. To extract protein, the retinas were lysed in RIPA lysis buffer containing protease inhibitors. Equal amounts of total proteins extracted from the retinas were separated by sodium dodecyl sulphate-polyacrylamide gel electrophoresis (SDS-PAGE) and then blotted onto polyvinylidene fluoride (PVDF) membranes. After blocking, the membranes were incubated first with antibodies, respectively, as follows: Gal-1 antibody (0.1 *μ*g/ml, R&D system, MN, USA); galectin-3 antibody (0.1 *μ*g/ml, R&D system, MN, USA); a monoclonal rabbit antibody against Nrp-1 (neuropilin-1 rabbit mAb, 1 : 1000, Cell Signaling Technology, MA, USA); a monoclonal rabbit antibody against VEGFR2 (rabbit mAb, 1 : 500, Cell Signaling Technology, MA, USA); a monoclonal rabbit antibody against pVEGFR2 (pVEGFR2 rabbit mAb, 1 : 500, Cell Signaling Technology, MA, USA); and a monoclonal rabbit antibody against *β*-actin (*β*-actin rabbit mAb, 1 : 1000, Cell Signaling Technology, MA, USA). After washing with TBS-T 3 times, the PVDF membranes were then incubated with horseradish peroxidase- (HRP-) conjugated goat anti-rabbit or donkey anti-goat IgG (1 : 5000, Jackson ImmunoResearch Laboratories, PA, USA) for 90 min at room temperature. The protein bands were visualized with chemiluminescence and analyzed with ImageJ.

### 2.7. Statistical Analysis

All data were presented as mean ± standard deviation (SD). Group differences were compared by one-way ANOVA with Bonferroni post hoc test for multiple comparisons. Data of the two groups were compared using the nonparametric Student's *t*-test. *P* values less than 0.05 were considered statistically significant.

## 3. Results and Discussion

### 3.1. Gal-1 Is Upregulated in OIR Model

Gal-1 is a carbohydrate-binding lectin with one CRD and binds to *β*-galactosides. Gal-1 has been proved as a proangiogenic factor in cancer-related angiogenesis [10, 11]. A study found that the knockdown of Gal-1 in endothelial cells inhibits cell proliferation [23]. Only a few microvessels were observed in Gal-1 null mice [23]. However, the role of Gal-1 in ocular neovascular diseases was not profoundly investigated to our knowledge. OIR mouse model is useful at investigating RNV and its treatment [6]. Mouse pups were exposed to 75% oxygen from P7 to P12 and then returned to room air until P17. Central retinal vasculature growth is suppressed during hyper oxygen exposure (from P7 to P12). After returning to room air, neovascularization occurs at the border between avascular and vascular zones [5, 6].

**Figure 1:**
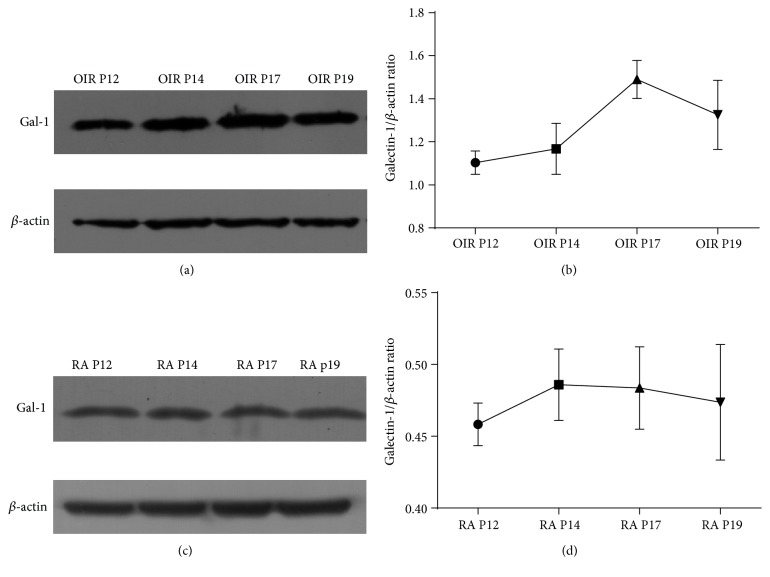
Western blot analysis of protein levels of Gal-1 from P12 to P19 in the RA group and the OIR group. (a) The OIR group. (b) Relative protein levels of Gal-1 in the OIR group indicated that Gal-1 reached the maximum at P17, when most of the RNV progression occurred at P17 (^∗∗∗^*P* < 0.001). (c) The RA group. (d) Western blot analysis showed that no significant change of Gal-1 were observed from P12 to P19 (*P* > 0.05). Protein bands were analyzed with ImageJ. Data were presented as the mean ± SD of three independent experiments.

**Figure 2:**
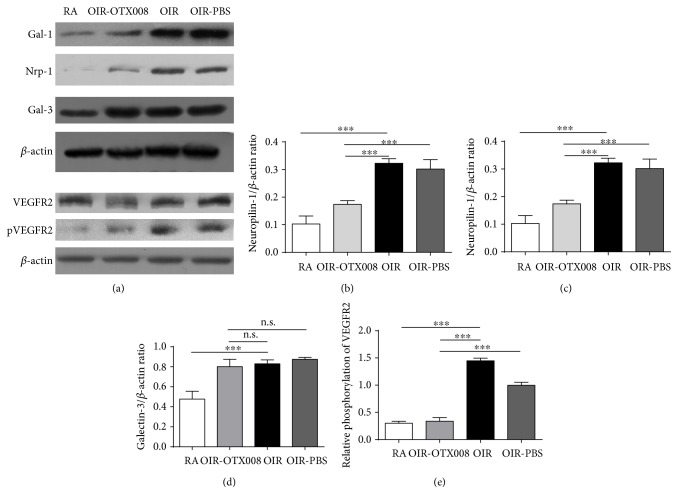
Western blot analysis of relative protein levels at P17. (a) Protein bands of Gal-1, Nrp-1, Gal-3, VEGFR2, pVEGFR2, and *β*-actin. (b–e) Relative protein levels of Gal-1, Nrp-1, Gal-3, and pVEGFR2. Protein level of Gal-1 was significantly inhibited in the OIR-OTX008 group than in the OIR group and in the OIR-PBS group, indicating the inhibitory function of intravitreal injection of OTX008. Protein level of Gal-3 was not affected by OTX008 compared to the OIR and OIR-PBS group (*P* > 0.05), indicating the specialty of OTX008 on Gal-1. In addition, Nrp-1 and pVEGFR2 were decreased. RA group versus OIR group, ^∗∗∗^*P* < 0.001; OIR-OTX008 group versus OIR group, ^∗∗∗^*P* < 0.001, OIR-OTX008 group versus OIR-PBS group, ^∗∗∗^*P* < 0.001; protein bands were analyzed with ImageJ. Data were presented as the mean ± SD of three independent experiments.

At first, we investigated the protein levels of Gal-1 in both the RA group and the OIR group at P12, P14, P17, and P19. Since most of the progression of RNV occurred at P17 in OIR, we found that the protein levels of Gal-1 reached the maximum at P17 in the OIR group, from P12 to P19 (*P* < 0.05; Figures 1(a) and 1(b)). In the RA group, no significant change of Gal-1 was observed from P12 to P19 (*P* > 0.05; Figures 1(c) and 1(d)). At P17, Gal-1 was remarkably overexpressed in OIR compared to RA control (*P* < 0.05; Figures 2(a) and 2(b)).

**Figure 3:**
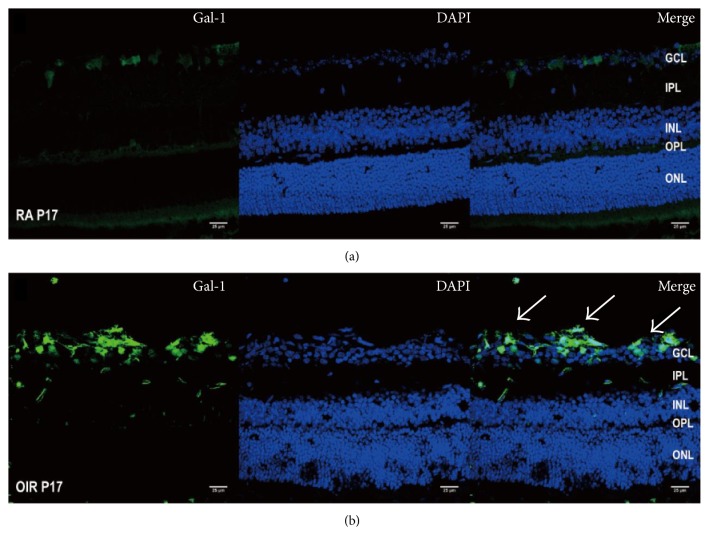
Location of Gal-1 in OIR. (a) RA group. (b) OIR group. At postnatal day 17 (P17), Gal-1 labeling of cryosection shows increased staining of Gal-1 antibody (green), mainly in the ganglion cell layer (GCL) and inner nuclear layer (INL) in OIR. Magnification 400x, scale bar = 25 *μ*m.

**Figure 4:**
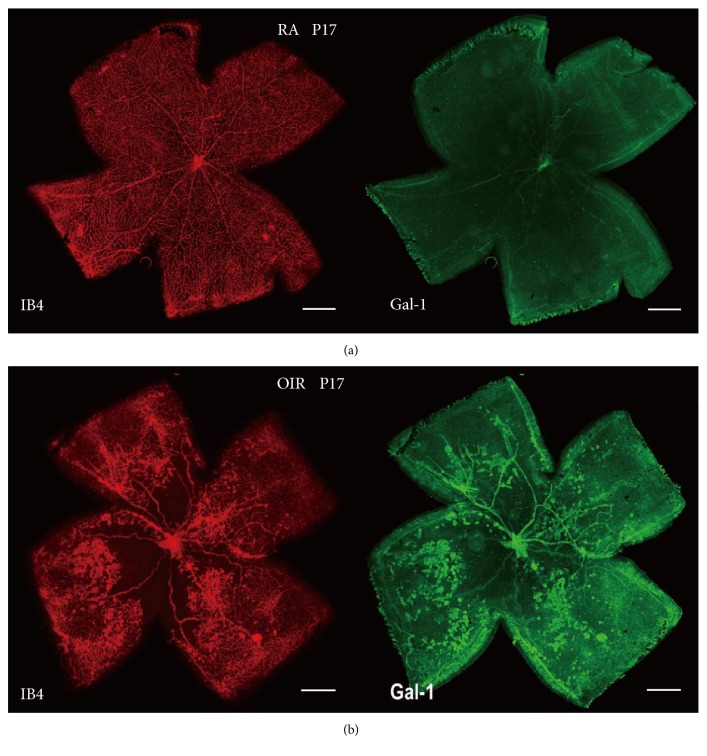
Distribution of Gal-1 in OIR at P17. (a) RA group. (b) OIR group. Whole-mount staining indicated that Gal-1 (*green*) was markedly enriched in the midperipheral area of the retina, which was consistent with the retinal neovessels (*red*) in OIR. Magnification 40x, scale bar = 500 *μ*m.

Besides, we investigated the distribution and location through immunofluorescence in RA and OIR. Cryosection of Gal-1 staining was present at both the RA and OIR groups, indicating the pathophysiological role of Gal-1. However, in the OIR group, the marked enrichment of Gal-1 staining at the ganglion cell layer (GCL) and inner plexiform layer (IPL) strongly suggested that Gal-1 was increased in OIR, in response to retinal ischemia (Figure 3). Meanwhile, whole-mount double staining of Gal-1 and isolectin B4 indicated that Gal-1 was obviously enriched in the midperipheral area of the retina, which was consistent with the retinal neovessels in OIR (Figure 4).

These results were similar to the previous reports which proved that Gal-1 plays an important role in tumor angiogenesis [10, 12]. Gal-1 has been implicated in several biological processes, such as cell adhesion, proliferation, apoptosis, and metastasis in vitro [24]. Our data further imply that Gal-1 is also involved in the course of retinal neovascularization in vivo.

### 3.2. Intravitreal Injection of OTX008 Decreases the Protein level of Gal-1

The preliminary outcome encourages us to further investigate the inhibition of Gal-1 on RNV. Suppression of Gal-1, such as blockade of CRD by oligosaccharides and derivatives or specific antibodies, indicated a promising inhibitory effect on tumor proliferation, invasion, and angiogenesis [15, 25]. Several Gal-1 targeting compounds have been produced recently. The most effective one is anginex, which interacts with Gal-1 specifically and inhibits tumor growth and angiogenesis in plenty of tumors [25]. OTX008, a novel calixarene derived from anginex, binds to Gal-1 specifically on the side back face [18]. Recent work indicated that OTX008 selectively inhibits Gal-1 and hinders cancer cell proliferation, invasion, and tumor angiogenesis as well [17]. The previous study showed that OTX008 could decreased Gal-1 protein level in a time-dependent manner in SQ20B cells, while not modulating galectin-3 (Gal-3) protein levels in SQ20B cells [18].

**Figure 5:**
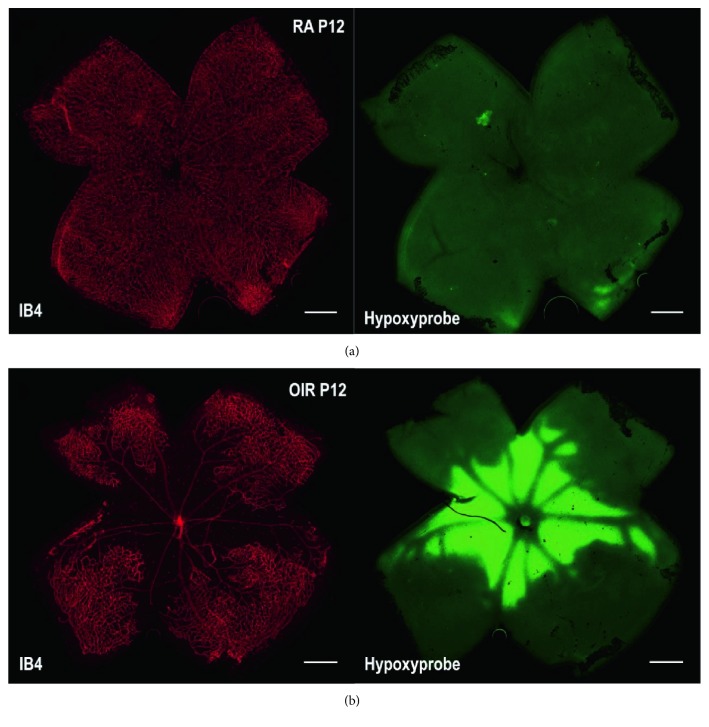
Retinal hypoxic condition in OIR at P12. (a) RA group. (b) OIR group. Whole-mount retinas were stained by isolectin B4 (*red*) and Hypoxyprobe (HP, *green*). Hypoxic areas and nonperfused zones were presented in the central retina in the OIR group, while no hypoxia was found in the RA group. Magnification 40x, scale bar = 500 *μ*m.

In our study, OTX008 was administered via intravitreal injection, to suppress the function of Gal-1 in OIR. Intravitreal injection has less adverse reactions than systemic administration and is consistent with clinical anti-VEGF therapies of treating retinal and choroidal neovascular diseases [9]. In our preliminary study, we found that the retinas from the OIR group were in a severe hypoxic condition 90 min after returning to room air at P12 (Figure 5). In OIR at P17, five days after injection of OTX008, western blot analysis was used to examine the protein levels of Gal-1. The outcome showed that Gal-1 was significantly decreased from the OIR-OTX008 group compared to those from the OIR group and the OIR-PBS group (*P* < 0.05; Figure 2), indicating the inhibitory role of OTX008, which is consistent with the previous study in vitro [18]. In addition, we also found that the protein level of Gal-3 was not altered after OTX008 injection (Figures 2(a) and 2(c)), implying the specialty of OTX008.

### 3.3. OTX008 Reduced the Number of Preretinal Neovascular Cells

**Figure 6:**
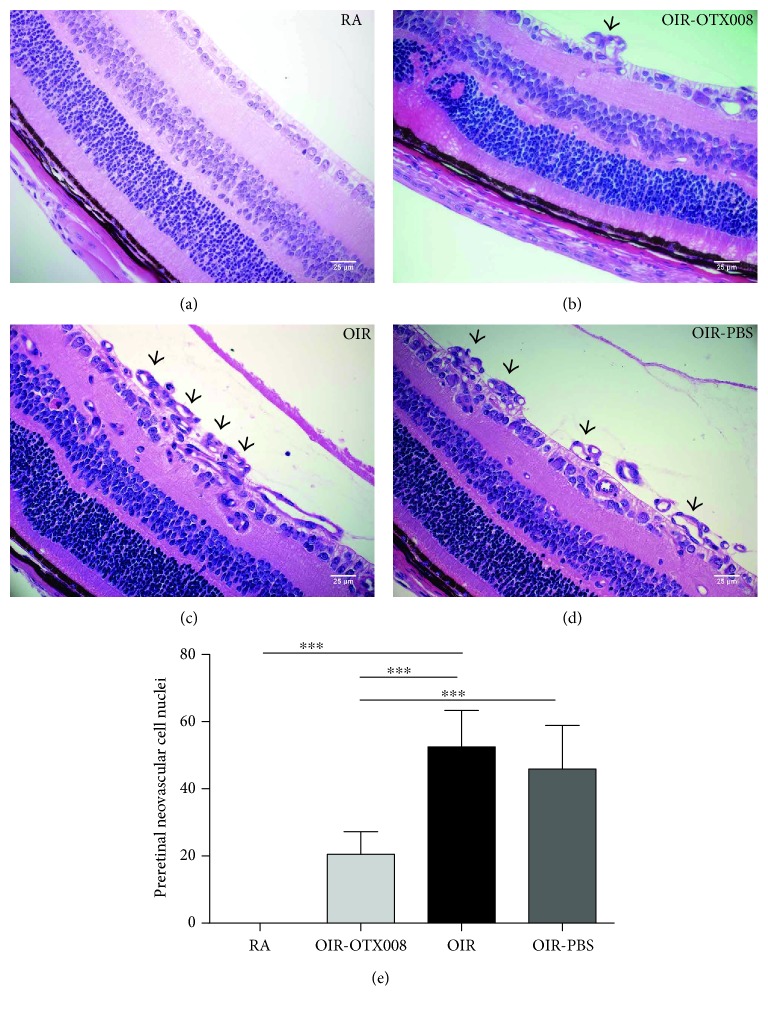
OTX008 reduced the number of preretinal neovascular cells. (a) RA group. (b) OIR-OTX008 group. (c) OIR group. (d) OIR-PBS group. At P17, sections from four groups were stained with H&E. *Black arrows* indicate the preretinal neovascular cells on the vitreous side of internal limiting membrane. Magnification 400x, scale bar = 20 *μ*m. (e) Quantification of preretinal neovascular cells. Quantification analysis revealed that intravitreal injection of OTX008 reduced the number of neovascular cells in the OIR-OTX008 group compared to those in the OIR group and in the OIR-PBS group. RA group versus OIR group, *t* = 11.51, ^∗∗∗^*P* < 0.001; OIR-OTX008 group versus OIR group, *t* = 7.018, ^∗∗∗^*P* < 0.001, OIR-OTX008 group versus OIR-PBS group, *t* = 5.565, ^∗∗∗^*P* < 0.001; data were presented as the mean ± SD, *n* = 8 per group.

Quantification of preretinal neovascular cell nuclei is a useful method of evaluating OIR model. Nuclei from new aberrant vessels and vessel profiles could be distinguished from other structures and could be counted [5, 19]. In our work, H&E staining was used to observe and count the number of preretinal neovascular cell nuclei on the vitreous side of the internal limiting membrane (Figure 6). The results clearly showed that the number of preretinal neovascular cells from the OIR group was more than that from the RA group (Figure 6), conforming the successful establishment of OIR model. After intravitreal injection of OTX008, the number was significantly reduced from the OIR-OTX008 group compared to that from the OIR group and the OIR-PBS group (*P* < 0.05; Figure 6), indicating the antiangiogenic function of OTX008.

### 3.4. OTX008 Suppressed the Retinal Neovascularization

Smith et al. [5] produced a consistent, reproducible, and quantifiable OIR mouse model, which is able to quantify the RNV by whole-mount fluorescent staining by calculating the area of neovascular tufts and nonperfused zones [6]. Isolectin B4, as a marker of the total vascular network [26], was used in our research to label retinal vasculatures. Pimonidazole (Hypoxyprobe) has been extensively applied as a marker to detect the hypoxia of cancer both in vitro and in vivo, which was applied in our study to assess the retinal hypoxia [21].

**Figure 7:**
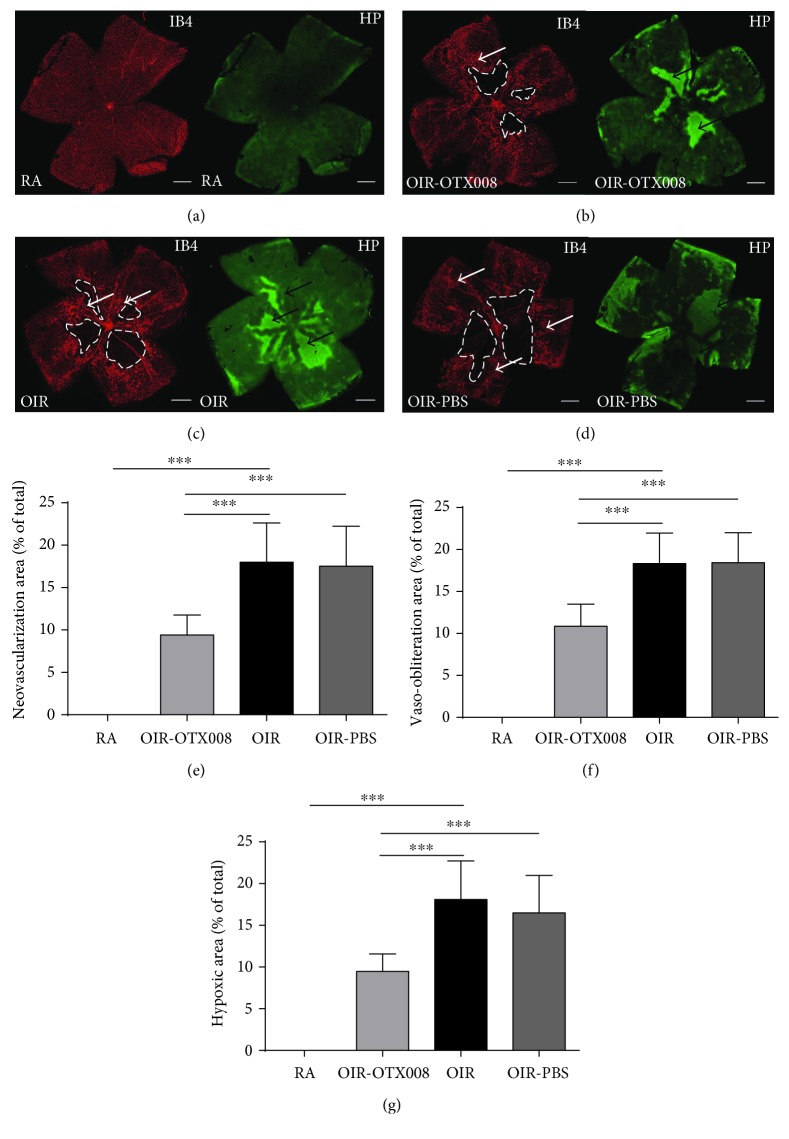
OTX008 suppresses retinal neovascularization (RNV) and attenuates retinal hypoxia. (a) RA group. (b) OIR-OTX008 group. (c) OIR group. (d) OIR-PBS group. At P17, whole-mount retinas were stained by isolectin B4 (*red*) and Hypoxyprobe (*green*). *White arrows* indicate neovascular tufts. *Black arrows* show hypoxic zones. *Irregular loops* depict VO areas. Magnification 40x, scale bar = 500 *μ*m. (e–g) Quantification of RNV, vaso-obliteration (VO), and hypoxic zones. RNV, VO, and hypoxia zones were decreased from the OIR-OTX008 group compared to the OIR group and to the OIR-PBS group. RA versus OIR, *t* = 11.46, *t* = 14.56, *t* = 11.94, ^∗∗∗^*P* < 0.001; OIR-OTX008 versus OIR, *t* = 5.459, *t* = 5.807, *t* = 5.686, ^∗∗∗^*P* < 0.001, OIR-OTX008 group versus OIR-PBS group, *t* = 5.170, *t* = 5.886, *t* = 4.639, ^∗∗∗^*P* < 0.001; data were presented as the mean ± SD, *n* = 10 per group.

Retinal flat-mount labeling showed obvious neovascular tufts emerging in OIR at the boundary between nonperfused zones and perfused zones at P17, while no RNV and vaso-obliteration areas were observed in the RA group (Figure 7). After administration of OTX008, the RNV area, VO area, and hypoxic zones were significantly decreased from the OIR-OTX008 group compared to those from the OIR and OIR-PBS groups (*P* < 0.05; Figure 7).

### 3.5. Effect of OTX008 on the Expression of Nrp-1 and pVEGFR2

**Figure 8:**
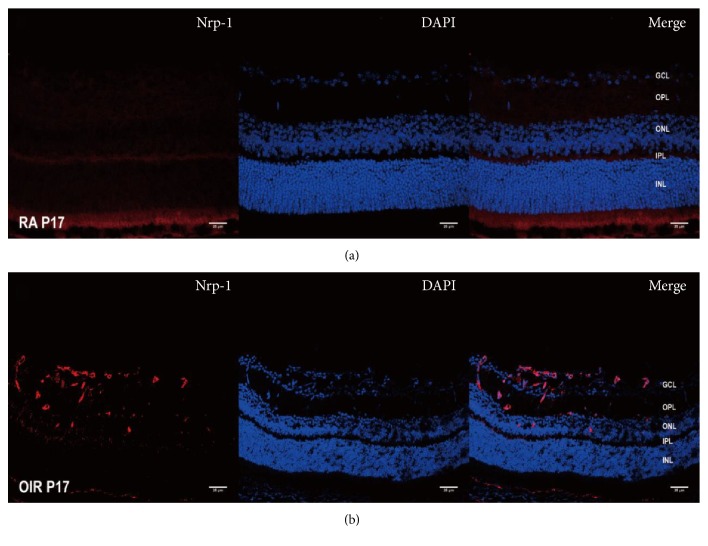
Immunostaining of Nrp-1 in OIR at P17. (a) RA group. (b) OIR group. Cryosection immunofluorescence showed increased staining of Nrp-1 antibody (red), mainly in the ganglion cell layer (GCL), inner plexiform layer (IPL), and inner nuclear layer (INL) in the OIR group, while much less staining of Nrp-1 is present in the RA group. Magnification 400x, scale bar = 25 *μ*m.

Our preliminary work also proved that Nrp-1 staining was markedly enriched from GCL and INL of the retinas in OIR (Figure 8). Western blot analysis in our study indicated that the protein levels of Nrp-1 and pVEGFR2 were increased in the OIR group compared to the RA group (*P* < 0.05; Figure 2), showing their proangiogenic role in RNV. However, both of them were reduced after intravitreal injection of OTX008 at P17 (*P* < 0.05; Figure 2).

**Figure 9:**
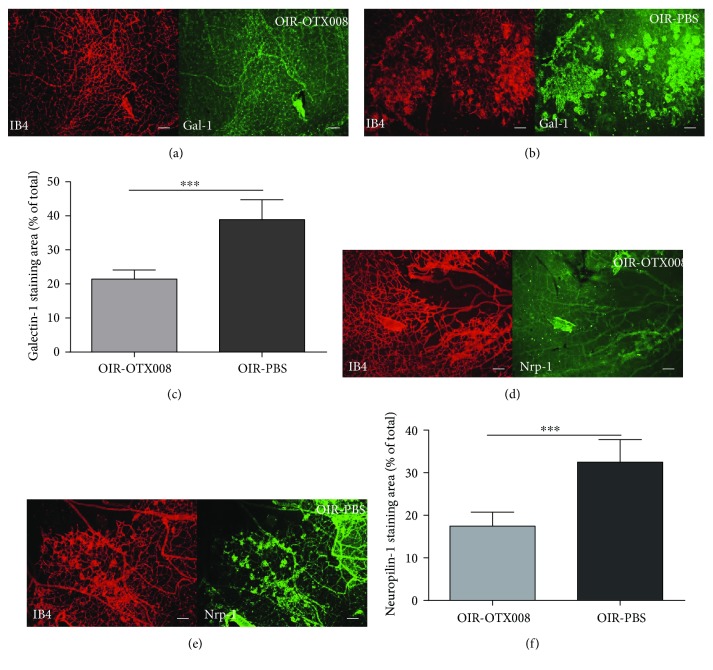
Quantification of Gal-1 and Nrp-1 staining at P17. (a) and (b) Whole-mount staining of Gal-1 and IB4 in the OIR-OTX008 group and the OIR-PBS group. (c) Quantification of Gal-1 staining showed that OTX008 injection significantly reduced the Gal-1+ area than in the OIR-PBS control group (^∗∗∗^*P* < 0.001). (d) and (e) Whole-mount staining of Nrp-1 and IB4 in the OIR-OTX008 group and the OIR-PBS group. (f) Quantification of Nrp-1 staining indicated that OTX008 injection significantly reduced the Nrp-1+ area than in the OIR-PBS control group (^∗∗∗^*P* < 0.001). Magnification 100x, scale bar = 100 *μ*m, data were presented as the mean ± SD, *n* = 8 per group.

In addition, quantification of whole-mount staining of Gal-1 and Nrp-1 was also performed to evaluate the effect of OTX008. The percentage of Gal-1 staining (Gal-1 + area/total retina) was significantly reduced in the OIR-OTX008 group than in the OIR-PBS group (*P* < 0.05; Figure 9). The percentage of Nrp-1 staining (Nrp-1 + area/total retina) was also significantly lower in the OIR-OTX008 group than in the OIR-PBS group (*P* < 0.05; Figure 9). This observation suggests that OTX008 reduced the level of Gal-1 and Nrp-1.

Nrp-1, as a neuronal receptor, mediates repulsive growth cone guidance and acts in endothelial cells as a co-receptor with vascular endothelial growth factor receptors (VEGFRs) for VEGF. A previous study demonstrated that Gal-1 selectively bonded to Nrp-1 via CRD and enhanced VEGFR2 phosphorylation [27]. Another report revealed that surface glycome of endothelial cell selectively mediated the binding to Gal-1, which relied on the recognition of VEGFR2 and then triggered VEGF-like signaling [11]. Croci et al. [11] found that the inhibition of intracellular or extracellular VEGF did not impact Gal-1 effects, implying these two molecules were regulated independently. Hsieh et al. [23] suggested that the co-expression of Nrp-1 and Gal-1 would be a novel interacting signal which modulates angiogenesis [23]. In our study, we also found that inhibition of Gal-1 by OTX008 decreased the protein level of Nrp-1, probably through preventing the phosphorylation of VEGFR2. These findings, which are consistent with previous work, open the way for further investigation.

## 4. Conclusion

Gal-1 was shown to be an important factor in oxygen-induced RNV. As a novel selective inhibitor of Gal-1, OTX008 was effective in reducing RNV and retinal hypoxia in a mouse model of OIR. The mechanism of its inhibitory function probably relies on the decrease of Nrp-1 and phosphorylation of VEGFR2. However, further work should be done to discover its pharmacokinetics and underlying mechanisms.

Ischemic retinopathy is one of the leading causes of blindness which is characterized by RNV. Potent antiangiogenic treatment with low toxicity and adverse reaction is required for ocular angiogenic diseases. In our study, OTX008 could be a promising compound in the strategy of treating RNV.
